# A Review of Conservative Surgical Approaches for Managing Placenta Accreta Spectrum

**DOI:** 10.7759/cureus.81551

**Published:** 2025-03-31

**Authors:** Sudhanshu K Rath, Asima Das, Mohini Mohini

**Affiliations:** 1 Obstetrics and Gynaecology, Kalinga Institute of Medical Sciences, Bhubaneswar, IND

**Keywords:** conservative surgery, placenta accreta spectrum (pas), prophylactic aortic balloon occlusion (pabo), uterine artery embolisation (uae), uterus preservation

## Abstract

The placenta accreta spectrum (PAS) was previously called the morbidly adherent placenta. It involves a range of pathological adherence of the placenta, including placenta increta, placenta percreta and placenta accreta, depending on the depth of invasion of anchoring villi into the myometrium and beyond. This spectrum of disorder is becoming a frequently encountered problem as a consequence of rising caesarean rates all over the world. Hysterectomy during caesarean delivery (CD) has been the conventional management of PAS. However, associated complications have prompted conservative surgery at the first sitting, with or without interventional radiology. The aim is to reduce blood loss and conserve the uterus if possible. It was decided to review the available literature on this emerging topic. Using PubMed and Google Scholar, our search focused on articles published from 2004 onwards, utilizing search terms "Placenta Accreta Spectrum” OR "Adherent Placenta" and "Caesarean Delivery". The selection for review adhered to specified inclusion/exclusion criteria and finally focused on 50 articles. It was found that published work includes the following conservative approaches: first, to leave the placenta expecting autolysis and resolution; second, to leave the placenta with the intention of a delayed hysterectomy; and third, to resort to an intraoperative arterial occlusion and follow-up. Though the primary objective of reducing blood loss at initial surgery is usually achieved, the success of uterine preservation differs. The availability of a multidisciplinary team for the surgical management of PAS is an important factor to consider. Limiting the extent of surgery at the time of CD, combined with or without arterial embolization/ballooning/ligation, reduces blood loss and limits morbidity with the potential to preserve the uterus.

## Introduction and background

One of the most serious events that can occur during a caesarean delivery (CD) in a pregnancy with a history of previous caesarean section (CS) happens due to the placenta accreta spectrum (PAS). The rates of CS have been a cause of concern all over the globe. The World Health Organization (WHO) issued a statement in 2015 indicating that a rise in CS rates of up to 10% results in a decrease in maternal and neonatal deaths. However, beyond that rate, it confers no benefit regarding maternal mortality [1]. This cautionary tone was expected to arrest the trend but did not. Various justifications were advanced, the most important being that it is necessary to consider both short- and long-term maternal and perinatal morbidity as outcomes are also necessary, not maternal mortality alone. A lack of data prevented the inclusion of these and other outcomes in the WHO analysis. By 2021, the WHO took a step back and clarified that it does not recommend a specific rate for countries to achieve. Consequently, a liberal approach gained wind and contributed to further rise, including but not limited to medically unindicated CS like caesarean on maternal request [2].

It is estimated that, without meaningful efforts to reverse the trend, approximately 38 million women worldwide will give birth via CS annually by the end of the present decade, with the rate reaching 63.4% in Eastern Asia. Many countries in South Asia and Africa will face a complex scenario regarding morbidity and mortality due to the overuse of a procedure in suboptimal working conditions [3].

One of the significant contributors to morbidity and mortality in subsequent pregnancies following a caesarean is PAS, as the majority of PAS cases manifest after a prior CS, although this is not exclusively the case. A previous myomectomy scar and vigorous curettage following an abortion can also play a causal role. Adherent placenta, first described in 1937, refers to the invasion of placental trophoblasts into the myometrium and beyond [4]. It was formerly known as morbidly adherent placenta. PAS refers to a range of pathological adherence of the placenta. The most favoured hypothesis regarding the aetiology of PAS is that there is a defect at the endometrial-myometrial interface, which leads to a failure of normal decidualisation, allowing for abnormally deep placental anchoring villi and trophoblast infiltration. The scarred uterus is a predisposing factor. Three subtypes are described as accreta (adherent to the myometrium), increta (invading the myometrium) and percreta (invasion of uterine serosa and beyond) [5]. The decidualisation process at endomyometrium junction in a scarred uterus is usually considered to play a role [5,6].

Most of the global population lives in developing countries, and most deliveries occur there. However, when we look for data, we face the problem of a lack of meticulous record-keeping and reporting from such countries. Therefore, we rely on data from the developed world. In the 1950s, the incidence of PAS was reported to be 0.03 per 1,000 pregnancies. Recent epidemiological studies estimate that the incidence of PAS is between 0.79 and 3.11 in 1,000 pregnancies [7]. A meta-analysis of 16 studies showed there is an increased risk of the PAS after CS [8]. In cases of placenta previa in a post-caesarean pregnancy, the coexistence of adherent placenta is proportional to the number of previous CS. One of the studies reported that in women presenting with placenta previa and prior CS, the incidence of PAS rose up to 40% after three CS deliveries [9].

Encountering an adherent placenta at the time of delivery is fraught with dangers, more so if undiagnosed earlier. Problems are multiple. These include extensive haemorrhage, requirement of massive blood transfusion, decision-making on uterus preservation, future fertility and injury to adjacent organs, particularly to the urinary tract. Spontaneous rupture of the uterus with PAS has also been reported during pregnancy [10]. As a result, PAS becomes an important contributing factor to maternal mortality [11].

With a diagnosis of PAS having been made, elective CD without the removal of the placenta, followed by a hysterectomy in the same sitting, is the conventional surgery [12]. Hysterectomy may be total or subtotal, depending on the situation. Complications with this surgical management, such as immediate hysterectomy, are pronounced. The question arises whether there is any alternative to immediate hysterectomy. When the bleeding is uncontrollable and the placenta is partially separated, a hysterectomy may become unavoidable despite the associated complications. However, in cases where the bleeding is under control or can be controlled, or the placenta is not at all separated, there are other options [13]. It is also true that with the advancement of and routine use of imaging modality, antenatal diagnosis of PAS has become a reality [14]. Interventional radiology has also opened up the scope of reducing blood loss at the time of CD for such cases [15]. Thus, a conservative approach to initial surgery is becoming a possibility to consider.

The purpose of this review is to collate and analyse the publications on surgery done for PAS with a particular focus on a conservative approach at the time of CS, meaning, thereby, any sort of immediate hysterectomy.

## Review

Method

Literature Search

To extensively review the literature, we employed PubMed and Google Scholar. Our search focused on articles published from 2004 onwards, utilizing search terms "Placenta Accreta Spectrum” OR "Adherent Placenta" and “Caesarean Delivery".

Inclusion/Exclusion Criteria

Final selection for review adhered to specific inclusion criteria: 1) original research articles/review/case series, 2) available in English, 3) peer-reviewed, 4) accessible in terms of full-text and 5) published within the designated time frame.

Article Screened

**Figure 1:**
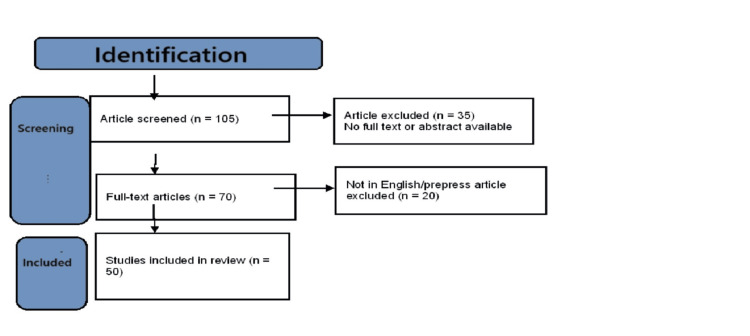
Article selection

Following the initial search, we pinpointed a total of 115 articles across the scrutinised databases. Subsequently, we removed duplicates (n = 10) and proceeded with an initial screening of titles and abstracts, leading to the exclusion of an additional 35 articles. Upon conducting a full-text screening of the remaining articles, 20 were further excluded for not meeting the inclusion criteria, as they were either unrelated to e-content or not focused on patient care. This process resulted in a final selection of 50 articles for the conclusive review, as shown in Figure 1.

Review

Once the diagnosis has been made as PAS, delivery of the baby by CS remains the preferred option. Essentially, three conservative approaches were undertaken at the time of CS. First, the placenta should be left without significant bleeding, with the expectation that autolysis and resolution will occur. Second, the placenta should be left in situ with the intention of performing a delayed hysterectomy, thereby reducing the risk of massive blood loss. This includes resorting to an intraoperative arterial occlusion process to minimise blood loss, with the intention of either hysterectomy or autolysis, depending on the future clinical course. Third, the partial resection and reconstruction of the uterus is indicated.

Group 1: Leaving Placenta In Situ for Resolution

As stated earlier, caesarean hysterectomy is considered the principal and preferred option for delivery for women with PAS [16,17]. However, alternative approaches, called "conservative management", have been developed with the primary objective of decreasing maternal morbidity. Preservation of fertility has been a secondary objective [18,19]. Leaving the placenta inside the uterus, awaiting complete resolution, intends to fulfill the primary objective. The extent to which it reduces blood loss, morbidity detected during follow-up, and achieves preservation of the uterus has been the subject of studies [19-24].

There was a multicentre study where PAS was diagnosed in 167 women [21]. They were managed by avoiding the removal of the uterus and leaving the placenta if haemostasis was achieved following the CD of the baby. During subsequent follow-ups, it was reported that a hysterectomy was avoided in 78% of cases, 42% needed transfusions and 6% had severe maternal morbidity. In those women where the uterus was preserved, the same author reported on positive reproductive outcomes in a subsequent article [22]. Accordingly, leaving the placenta in situ as a possible management strategy, particularly for women wishing to preserve their fertility, began to be an option [23-28]. However, only three small retrospective studies compared outcomes between women managed by either hysterectomy or leaving the placenta in situ after CD. Though the results favoured leaving the placenta, study designs had limitations, with a small sample size and single-centred study [29-31].

To overcome such a limitation, a large-scale multicentre study was designed by French researchers as an observational cohort study from a source population of 520,114 deliveries from 176 hospitals. It included women with PAS who had either a hysterectomy or a placenta left in situ during CD [32]. Women meeting the inclusion criteria were identified antenatally and included at delivery. Eighty-six women had conservative management, and 62 women had a caesarean hysterectomy for PAS during CD. The primary outcome was a transfusion requirement within six months postpartum. Secondary outcomes were other maternal complications within the same period. The authors used propensity-score weighting to account for potential indication bias. The conclusion drawn was that there is a lower risk of severe blood loss, but there is a higher risk of endometritis in patients undergoing a conservative approach.

This trial has encouraged further investigation of the scope of conservative management [33]. International Federation of Gynecology and Obstetrics guidelines on conservative management of PAS include such an approach and may result in wider adoption [34]. At the same time, the results of this study were not welcome in the United States, where multidisciplinary care is common, and the teams are comfortable with performing immediate hysterectomies, which has already led to reduced morbidity and mortality. The American College of Obstetricians and Gynecologists and the Society for Maternal-Fetal Medicine consensus statement on PAS recommends hysterectomy as standard management [35]. Differences in the organisation of the healthcare system may result in substantial differences in maternal outcomes between countries. In France, where the above PACCRETA study [32] was conducted, teams have experience in managing short and mid-term complications associated with a conservative approach. The requirement of close monitoring with scheduled visits during several months is possible to meet as women live not too far from the centre, taking responsibility for their care. To undertake an aggressive procedure, the availability of a multidisciplinary team is a must. With deeper placental invasion, the performance of the surgery and management by a multidisciplinary team are associated with improved maternal outcomes [36].

The risk of infection with the placenta left in situ is a reality. This likely complication should always be discussed with the patient for shared decision-making.

Group 2: Devascularisation and Delayed Hysterectomy

Hysterectomy in the initial sitting is associated with massive blood loss and consequent complications. Allowing some time for involution and reduced vascularity and undertaking a hysterectomy later might be prudent. Placement of an occlusive device or embolisation of the feeding artery at initial surgery can be of further assistance.

A planned placement of a uterine artery catheter prior to surgery, followed by the performance of a CD of the baby and immediate uterine artery embolisation (UAE) during surgery, reduces blood loss. An elective delayed hysterectomy after three to four weeks as part of the plan completes the surgical management. This approach of delayed hysterectomy might be a reasonable option in most cases of placenta percreta. In a small series, delayed hysterectomy was possible only in 50% of cases [37].

In a larger prospective case series of subjects from 2005 to 2018 with suspected placenta percreta, 22 cases underwent UAE while doing CD. A hysterectomy after five weeks was part of the plan. It was reported that in 17 cases (77.3%), a scheduled delay of more than five weeks could be achieved. The blood transfusion requirement was lower. They concluded that the UAE, at the time of elective CS, was safe and feasible. A delay of five weeks for a hysterectomy appears to be possible, but not in all cases, as an unscheduled hysterectomy was needed in 22.7% [38]. Therefore, patient selection and close follow-up are essential.

In another case series, prophylactic UAE was done just after caesarean to minimise blood supply to the uterus and placental bed. Thereafter, removal was attempted in accrete, and the placenta was left inside the uterus in deeper invasion. Placenta left in situ cases received methotrexate 50 mg/week. The decision to do a hysterectomy was made according to the clinical course. With this approach, authors reported overall decreased blood loss and the requirement of hysterectomy in one of the four cases during attempted removal and two of the seven cases during the follow-up period. Intraoperative embolisation of bilateral feeding vessels reduces the risk of massive haemorrhage and increases the chance of preservation of the uterus [39].

Occlusion of feeding vessels by a balloon device during the surgery to reduce bleeding has also been attempted. The device is placed preoperatively and inflated after the delivery of the baby. The procedure, when done to the aorta, is prophylactic balloon occlusion (PABO) of the infrarenal abdominal aorta. Its use in PAS was reported in 2012. In a retrospective study, data from 268 cases were analysed. Performing a hysterectomy was avoided in 230 cases [40]. In low-resource settings, the use of an aorta clamp has been reported in 33 cases of PAS. It can be used easily to occlude the abdominal aorta even without the need for any retroperitoneal dissection [41]. However, the objective of the study was to decrease blood loss, not avoid hysterectomy. This objective was met, and hysterectomy was avoided in four out of 33 cases. Prophylactic balloon occlusion of internal iliac (POIIA) was studied in a meta-analysis of 29 articles with a total sample size of 2,365 patients with POIIA as the intervention in the study group. The results of this study showed that POIIA has the benefits of reduced intraoperative blood loss and reduced hysterectomy [42]. Only in two studies was POIIA not found to offer a significant advantage in patients with PAS [43,44].

Another point of interest is that, instead of ballooning, for which interventional radiology support is essential, the internal iliac can be ligated at surgery, thereby reducing dependence. A study of 79 cases compared the two approaches. It was observed that women in whom endovascular balloons were placed had procedure-related complications in 10% of cases, besides an overall increased total procedure time. There was no difference in blood loss compared to those undergoing surgical ligation [45]. Similarly, instead of embolisation of the uterine arteries, some researchers have reported ligation of the trunks in a low-resource setting. A series of 63 cases was reported. The surgical procedure involved the division of the round ligaments, the opening of the broad ligament, the dissection of the bladder from the lateral aspect, and direct bilateral uterine artery ligation below the placental bed. Only five patients needed hysterectomy [46]. At the same time, taking interventional radiology a step further by its proponents, it has been reported that intra-operative POIIA combined with post-operative UAE may be an effective strategy in conservative management [47].

Blood loss is reduced with any of the approaches in this group, which is the primary aim. Conservation of the uterus is not the intent, as hysterectomy has only been deferred. The risk of infection during the intervening period is expected, and close follow-up is essential.

Group 3: Partial Resection and Reconstruction of Uterus

Some researchers have conceived that, instead of leaving the deeply adherent placenta, it may be possible to excise the adherent placenta along with part of the uterus and reconstruct it. In a 2004 study, it was reported that haemostasis could be achieved by vascular ligature and uterine compression after the resection of part of the uterine wall with the adherent placenta. The procedure involved the repair of the defect on the anterior wall with a myometrial suture. They also used fibrin glue and a polyglycolic mesh. A non-adherent cellulose layer was applied to this reconstruction. Hysteroscopy and MRI were performed to check the reconstructed uterus after 90 days [48]. They could preserve the uterus in 50 out of 68 cases. A triple-P procedure was described by Chandraharan et al. [49], which involved three main steps: placental upper edge localisation, pelvic devascularisation by UAE and placental non-separation. The placenta with adherent myometrium was excised, followed by repair of the defect. It was proposed as a safe and effective alternative to conservative management. A modified triple-P approach was also tried. In this modification, ligation of the internal iliac vessels was resorted to instead of embolisation, which may be more suitable when a radiologically guided procedure is not available. The requirement of hysterectomy in women who had the modified triple-P approach was 14.28% [50]. This conservative stepwise surgical approach for the management of PAS has been followed by other researchers, and a success rate of 80.64% has been achieved [51]. Partial resection and reconstruction approaches have been the subject of a multicentre study recently involving nearly 600 women with PAS, where the preservation of the uterus was reported in 80% of cases [52].

In another interesting series of 68 cases from Vietnam, Thi Pham et al. advocated Modified One-Step Conservative Uterine Surgery (MOSCUS). In addition to uterine artery ligation, they addressed the haemorrhage issue with cervical tourniquets and transverse B lynch. Resection of a portion of the anterior uterine wall with unseparated placenta and uterine reconstruction was done. The average estimated blood loss was 987 mL, and they reported preservation of the uterus in 64 cases [53]. Earlier, researchers from China also reported partial myometrium resection and uterine preservation during the primary procedures in a series of 62 cases. PABO was employed in this series to minimise blood loss [54]. Only one case required a hysterectomy. Intraoperative mean blood loss was 1,377 mL.

As stated earlier in this article, in many ways, the economic level of a country or geographical area influences the management approach. Thus, the practice preference for PAS generates controversy based on the resource setting. A recent meta-analysis was conducted where 11 observational studies from middle-income countries were included, with a total of 1,600 patients. It was concluded that besides preservation of the uterus, conservative management is associated with decreased blood loss and lower risks of adjacent organ injury, hospitalisation duration, operative time and intensive care unit admission. There were no significant differences in risks like coagulopathy or thromboembolism [55]. With the focus on conservative surgery and uterine preservation, some researchers have made efforts to provide a prediction model for the success of uterine preservation, which may assist decision-making [56].

When writing, data on the long-term effects of uterine reconstruction are not available. Intra-abdominal adhesions following extensive surgery and chronic pelvic pain are possible.

## Conclusions

PAS is frequently encountered with rising CS rates. Diagnosis is not difficult with available imaging modalities. It is the management that throws many challenges. Traditional surgery of caesarean hysterectomy is time-tested, definitive and still the recommended practice. However, massive bleeding is inevitable. Loss of reproductive and menstrual function may not be acceptable to all. Therefore, many researchers have attempted conservative surgery at the first sitting. Limiting the extent of surgery at the time of CD means preservation of the uterus at the initial setting. Combined with or without arterial embolisation/ballooning/ligation, blood loss is reduced, and morbidity is limited. The intentions have been either leaving the placenta for auto-resolution or subsequent delayed elective hysterectomy. In both situations, close follow-up is essential, as an unscheduled hysterectomy may be necessary. It must be remembered that morbidity like infection with placenta in situ is a factor to reckon. MOSCUS may sound interesting, but a reconstructed uterus can have multiple adhesions and source of chronic pelvic pain in the long term. Operative skill in undertaking such surgery, in fact, any PAS surgery, is of paramount importance. It must always be undertaken by a senior experienced obstetrician while ensuring the involvement of an intervention radiologist, urologist, anaesthetist, and critical care and transfusion specialist.

Strength of this review lies in collating the literature on an evolving alternative. Limitation is confining the search to two data bases. Area of future research can be studies on long term morbidity and future fertility outcome where uterus was preserved. Refinement of prediction model for success may assist decision making and promote incorporation of uterine preservation procedures in the institutional PAS protocols.
